# Cutaneous adverse events following CAR T-cell therapy in hematologic malignancies

**DOI:** 10.1038/s41408-026-01471-5

**Published:** 2026-03-12

**Authors:** Elvira Umyarova, John Sharp, Charles Pei, William Pellegrino, Qiuhong Zhao, Nathan Denlinger, Timothy Voorhees, Marcos De Lima, Narendranath Epperla

**Affiliations:** 1 Division of Hematology, The Ohio State University Comprehensive Cancer Center, Columbus, OH USA; 2 Department of Medicine, The Ohio State University, Columbus, OH USA; 3 Division of Hematology and Hematologic Malignancies, Huntsman Cancer Institute, University of Utah, Salt Lake City, UT USA

**Keywords:** Health care, Medical research

## To the Editor

Chimeric antigen receptor (CAR) T-cell therapy has transformed outcomes for patients with relapsed or refractory (R/R) hematologic malignancies [1], particularly diffuse large B-cell lymphoma (DLBCL), mantle cell lymphoma (MCL), and B-acute lymphoblastic leukemia (B-ALL). While cytokine release syndrome and neurotoxicity are well-characterized toxicities of CAR T-cell therapy, data on associated cutaneous adverse events (AEs) are limited [2–4]. Hence, we sought to evaluate the incidence, type, and timing of cutaneous AEs following CAR T-cell therapy.

This is a single center retrospective cohort study of patients with of R/R B-cell lymphoma, B-ALL, or multiple myeloma who were treated with commercial CAR-T products (axicabtagene ciloleucel, tisagenlecleucel, lisocabtagene maraleucel, brexucabtagene autoleucel, or idecabtagene vicleucel) between January 1, 2016, and September 1, 2022, at Ohio State University. Medical records were reviewed to determine the incidence and severity of cutaneous AEs following CAR T-cell therapy. Treating oncologist documentation, dermatology consultation notes, and skin biopsy findings were systematically assessed. Two authors (C.P. and W.P.) independently adjudicated each cutaneous AE and classified its likelihood of association with CAR T-cell therapy as low or high. High likelihood of association was defined as lack of alternative etiologies for cutaneous AE. Cases with uncertain attribution were reviewed by a third author (E.U.) to reach consensus. Only cutaneous AEs deemed to have a high likelihood of association with CAR T-cell therapy were included in the analysis. Cutaneous AEs were graded per CTCAE v5.0. Cutaneous AEs were categorized based on the time of onset following CAR-T infusion as early-onset (within 30 days), intermediate-onset (31–180 days), and late-onset (beyond 180 days). The study was approved by the institutional review board at Ohio State University and was conducted in compliance with the Declaration of Helsinki. As this was a retrospective study, informed consent was waived.

Among the 246 patients included in the study, 97% (*n* = 228) were treated for R/R B-cell lymphomas and B-ALL, and 3% (*n* = 18) for R/R multiple myeloma. Median follow-up was 576 days (range 16 – 2041 days). Among these, cutaneous AEs of high likelihood of association with CAR T-cell therapy occurred in 23 patients and were the population of interest. An additional 34 patients had cutaneous AEs deemed to be of low likelihood of association with CAR T-cell therapy. Table 1 outlines the characteristics of patients with cutaneous AEs following CAR T-cell therapy. The median age was 66 years (range 54–78), with 65% males. The median number of lines of therapy prior to CAR-T was 3 (range, 2-7). Five (22%) patients received prior autologous hematopoietic stem cell transplant (HSCT), and 1 (4%) patient received prior allogeneic HSCT. Most patients (*n* = 21, 91%) had chemorefractory disease at the time of CAR-T infusion. Of note, none of the patients who received CAR T-cell therapy for multiple myeloma developed cutaneous AEs in our cohort.

**Fig. 1:**
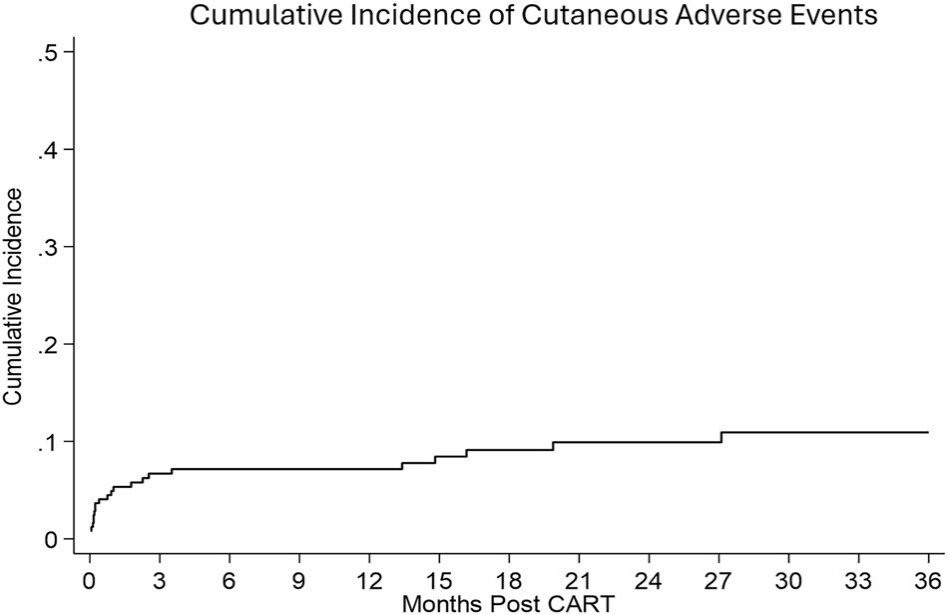
Cumulative incidence of cutaneous AEs following CAR T-cell therapy.

The cumulative incidence of cutaneous AEs was 11% by 36 months post-CAR-T (Fig. 1). The most common cutaneous AEs were maculopapular rash (*n* = 9, 39%), pruritic macular rash (*n* = 5, 22%), and bullous dermatitis (*n* = 2, 9%). In the two patients that experienced bullous dermatitis, it was observed as early onset AE presenting as blistering rash in one and intermediate-onset rash presenting as inflammatory bullae on both lower extremities in the other. Skin biopsies from both patients showed non-specific findings and lesions resolved without any intervention. Less frequent AEs included acneiform rash, skin erythema and hyperalgesia, mucocutaneous ulcer, and other skin and subcutaneous tissue disorders (Table S1). All cutaneous AEs were grade 1 (*n* = 16, 70%) or grade 2 (*n* = 7, 30%). In total, 6 (26%) patients received dermatologic consultation and 2 (9%) underwent skin biopsy.

The timing of the onset of cutaneous AEs varied considerably (Table S1). The majority of cases (57%) were early-onset, possibly linked to acute inflammatory or cytokine-mediated mechanisms. An additional 22% occurred between 30 and 180 days (intermediate onset), which may reflect evolving immune reconstitution or delayed hypersensitivity phenomena. Interestingly, 22% of cases developed late cutaneous AEs, beyond 180 days post-infusion, suggesting a possible role of chronic immune activation. The distribution of type of cutaneous AE over time did not differ significantly (*p* = 0.43 Table S2). Three patients (13%) experienced multiple distinct cutaneous AEs, however, only the first episode following CAR T-cell therapy was included in the analysis. One (4%) patient developed cutaneous AE after progression of their underlying malignancy following CAR T-cell therapy but prior to initiation of any subsequent therapy. These findings underscore the importance of long-term follow-up, as cutaneous AEs may occur in a delayed fashion, even after resolution of systemic inflammatory toxicities and progression of underlying disease. Among affected patients, seven of twenty-three (30%) were treated with topical corticosteroids, such as hydrocortisone or triamcinolone, and two patients (9%) received topical antibiotic therapy. On univariable analysis, an increasing number of prior lines of therapy was associated with a lower risk of cutaneous AEs following CAR T-cell therapy (HR 0.77 [95% CI 0.60, 0.99] *p* = 0.04, Table S3).

We found no cutaneous AEs following CAR-T therapy in multiple myeloma, though these occurred in 10-14% of patients in registrational trials [5]. The reasons for this remain unclear, but the relatively small sample size (*n* = 18) and potential differences in disease biology may have contributed to this finding. Larger studies are needed to further evaluate this observation.

In this study, we found that cutaneous AEs are not uncommon following CAR-T therapy, occurring in approximately 1 in 10 patients. This is in line with rates of cutaneous AEs observed in registrational trials for commercially available CAR-T products, ranging from 8 to 31% [5–9]. The cutaneous AEs are mostly low-grade and predominantly occurred early following CAR T-cell therapy. However, late occurrences were also noted. The predominance of maculopapular rash parallels patterns observed with other immunotherapies, raising the possibility of T-cell mediated mechanisms. The occurrence of bullous dermatitis underscores the potential for rare but clinically significant immune-mediated pathology [10].

The observed inverse association between the number of prior therapies and the risk of cutaneous AEs may reflect greater immune competence in patients with less prior treatment exposure, enabling a more robust cutaneous inflammatory response; however, further studies are needed to validate and elucidate the mechanisms underlying this association.

Limitations of this study include the relatively small sample size, the possibility of unrecognized alternative etiologies for cutaneous AEs, and an underestimation of the total number of cutaneous AEs due to the real-world nature of the study. In addition, we were unable to determine the incidence of rashes in comparable patients not receiving CAR T-cell therapy, as the study cohort was limited to CAR T-cell recipients. Notwithstanding these limitations, the findings provide clinically meaningful insights for patients, clinicians, and researchers.

To our knowledge, this study represents one of the first systematic evaluations of cutaneous AEs following CAR T-cell therapy and underscores the need for prospective assessment of cutaneous toxicities in CAR T-cell clinical trials. Early referral to dermatology for persistent or severe presentations, along with continued vigilance for late-onset toxicities, is warranted. Given the expanding role of CAR T-cell therapy across disease settings, incorporation of dermatologic surveillance and supportive care strategies is essential. Further studies are needed to identify factors predictive of cutaneous AEs following CAR T-cell therapy and to elucidate the underlying mechanisms and pathophysiology of these toxicities.

**Table 1:** Characteristics of patients with cutaneous adverse events after CAR T-cell therapy.

Patient characteristics	*N* = 23 (%)
**Median age at CAR-T in years (range)**	66 (54–78)
**Median number of lines of therapy prior to CAR-T (range)**	3 (2–7)
**Sex**	
Male	15 (65)
Female	8 (35)
**Disease type**	
DLBCL	17 (74)
Transformed lymphoma	5 (22)
MCL	1 (4)
**Type of CART product**	
Axicabtagene ciloleucel	10 (44)
Tisagenlecleucel	11 (48)
Lisocabtagene maraleucel	1 (4)
Brexucabtagene autoleucel	1 (4)
**Prior auto-HCT**	5 (22)
**Prior allo-HCT**	1 (4)
**Disease status pre-CAR-T**	
PR	2 (9)
SD	3 (13)
PD	18 (78)
**Stage**	
II	4 (17)
III or IV	19 (83)

*CART* chimeric antigen receptor T-cell therapy, *B-ALL* B-cell acute lymphoblastic leukemia, *DLBCL* Diffuse large B-cell lymphoma, *MCL* mantle cell lymphoma, *auto-HCT* autologous hematopoietic cell transplantation, *allo-HCT* allogeneic hematopoietic cell transplantation, *PR* partial response, *SD* stable disease, *PD* progressive disease.

## Supplementary information

Supplemental Appendix

## Data Availability

Data is available upon request to the corresponding author as permitted by the IRB.
