# Supplementary material for: Cutaneous adverse events following CAR T-cell therapy in hematologic malignancies
Source: Blood Cancer J. 2026 Mar 12;16(1):29. doi: 10.1038/s41408-026-01471-5 (PMC12982786; doi:10.1038/s41408-026-01471-5)
Supplement: Supplementary file 1 — Supplemental Appendix [file 41408_2026_1471_MOESM1_ESM.docx]

**SUPPLEMENTAL APPENDIX**

**Table of Contents** 1

**Type, grade, and onset of cutaneous adverse events following chimeric antigen receptor T-cell therapy:** Table S1 2

**Table S2: Distribution of types of cutaneous adverse event over time:** Table S2 3

**Univariable analysis of baseline characteristics and associated risk of developing cutaneous adverse event following chimeric antigen receptor T-cell therapy:** Table S3 4

**Table S1.** Type, grade, and onset of cutaneous adverse events following chimeric antigen receptor T-cell therapy

| **Cutaneous Adverse Effects** | **N=23 (%)** |
| --- | --- |
| **Type of reaction** |  |
| Maculopapular rash | 9 (39) |
| Pruritic macular rash | 5 (22) |
| Bullous dermatitis | 2 (9) |
| Acneiform rash | 1 (4) |
| Skin erythema and hyperalgesia | 1 (4) |
| Mucocutaneous ulcer | 1 (4) |
| Skin and subcutaneous tissue disorders – other* | 4 (17) |
| **Grade per CTCAE v5.0** |  |
| 1 | 16 (70) |
| 2 | 7 (30) |
| **Time of onset** |  |
| Early (≤ 30 days) | 13 (57) |
| Intermediate (30-180 days) | 5 (22) |
| Late (>180 days) | 5 (22) |

Abbreviations: CTCAE v5.0=Common Terminology Criteria for Adverse Events, version 5.0

*Others include – skin and subcutaneous tissue disorders not fitting into one of the categories. These include diffuse ecchymosis on the arms, legs and face and undifferentiated skin rash on legs.

**Table S2:** Distribution of types of cutaneous adverse event over time

| **Timing of onset** | **Early** (≤ 30 days) | **Intermediate** (30-180 days) | **Late** (>180 days) | **p-value** |
| --- | --- | --- | --- | --- |
| Maculopapular rash | 5 | 3 | 1 | 0.43 |
| Pruritic macular rash | 2 | 0 | 3 |  |
| Bullous dermatitis | 1 | 1 | 0 |  |
| Acneiform rash | 0 | 1 | 0 |  |
| Skin erythema and hyperalgesia | 1 | 0 | 0 |  |
| Mucocutaneous ulcer | 1 | 0 | 0 |  |
| Skin and subcutaneous tissue disorders – other* | 3 | 0 | 1 |  |

*Others include – skin and subcutaneous tissue disorders not fitting into one of the categories. These include diffuse ecchymosis on the arms, legs and face and undifferentiated skin rash on legs.

**Table S3**: Univariable analysis of baseline characteristics and associated risk of developing cutaneous adverse event following chimeric antigen receptor T-cell therapy.

| **Patient characteristics** | HR | 95% CI | | p-value |
| --- | --- | --- | --- | --- |
| **Age at CAR-T (in years)** | 1.02 | 0.99 | 1.05 | 0.15 |
| **Number of lines of therapy prior to CAR-T** | 0.77 | 0.60 | 0.99 | **0.04** |
| **Sex** |  |  |  |  |
| Female | Ref |  |  |  |
| Male | 1.20 | 0.49 | 2.95 | 0.68 |
| **Disease type** |  |  |  |  |
| DLBCL/HGBL/PMBCL | Ref |  |  |  |
| Richter's transformation | * |  |  |  |
| Transformed lymphoma | 1.08 | 0.36 | 3.26 | 0.89 |
| B-ALL | * |  |  |  |
| FL | * |  |  |  |
| MCL | 0.55 | 0.08 | 3.74 | 0.54 |
| MM | * |  |  |  |
| CLL | * |  |  |  |
| Burkitt | * |  |  |  |
| **Type of CART product** |  |  |  |  |
| Axicabtagene ciloleucel | Ref |  |  |  |
| Brexucabtagene autoleucel | 0.59 | 0.08 | 4.31 | 0.60 |
| Tisagenlecleucel | 1.33 | 0.56 | 3.18 | 0.52 |
| Lisocabtagene maraleucel | 3.85 | 0.64 | 23.09 | 0.14 |
| Idacabtagene vicleucel | * |  |  |  |
| **Prior autoSCT** | 0.47 | 0.16 | 1.36 | 0.16 |
| **Prior alloSCT** | * |  |  |  |

Abbreviations: AlloSCT=allogeneic stem cell transplant, autoSCT=autologous stem cell transplant, B-ALL=B-cell acute lymphoblastic leukemia, Burkitt=Burkitt’s lymphoma, CAR-T=chimeric antigen receptor T-cell therapy, CI=confidence interval, CLL=chronic lymphocytic leukemia, DLBCL=diffuse large B-cell lymphoma, FL=follicular lymphoma, HGBL=high grade B-cell lymphoma, HR=hazard ratio, MCL=mantle cell lymphoma, MM=multiple myeloma, PMBCL=primary mediastinal B-cell lymphoma.

*Insufficient events within this group to be considered in univariable analysis model.
